# Effect of trunk exercise upon lumbar IVD height and vertebral compliance when performed supine with 1 g at the CoM compared to upright in 1 g

**DOI:** 10.1186/s13102-022-00575-2

**Published:** 2022-10-07

**Authors:** D. Marcos-Lorenzo, T. Frett, A. Gil-Martinez, M. Speer, J. Swanenburg, D. A. Green

**Affiliations:** 1 School of Medicine of Autonomous, University of Madrid, 28029 Madrid, Spain; 2 Department of Aerospace Physiology, Institute for Aerospace Medicine, German Aerospace Center E.V. (DLR), 51147 Cologne, Germany; 3 Department of Physiotherapy, Centro Superior de Estudios Universitarios La Salle, Universidad Autónoma de Madrid, 28023 Madrid, Spain; 4 Space Medicine Team, European Astronaut Centre, European Space Agency, Linder Höhe, 51147 Cologne, Germany; 5 Integrative Spinal Research ISR, Department of Chiropractic Medicine, Balgrist University Hospital, UZH Space Hub Space Life Sciences, University of Zurich, Lengghalde 5, 8008 Zurich, Switzerland; 6 University of Zurich, Zurich, Switzerland; 7 Centre of Human and Applied Physiological Sciences, King’s College London, London, SE1 1UL UK; 8 KBRwyle GmbH, Albin Köbis Straße 4, 51147 Cologne, Germany

**Keywords:** Artificial gravity, Countermeasures, IVD, Exercise, Stiffness, Spine

## Abstract

**Background:**

Spinal unloading in microgravity is associated with stature increments, back pain, intervertebral disc (IVD) swelling and impaired spinal kinematics. The aim of this study was to determine the effect of lateral stabilization, trunk rotation and isometric abdominal exercise upon lumbar IVD height, and both passive and active vertebral compliance when performed supine on a short-arm human centrifuge (SAHC)—a candidate microgravity countermeasure—with 1 g at the CoM, compared to that generated with equivalent upright exercise in 1 g.

**Methods:**

12 (8 male) healthy subjects (33.8 ± 7 years, 178.4 ± 8.2 cm, 72.1 ± 9.6 kg) gave written informed consent. Subjects performed three sets of upper body trunk exercises either when standing upright (UPRIGHT), or when being spun on the SAHC. Lumbar IVD height and vertebral compliance (active and passive) were evaluated prior to SAHC (PRE SAHC) and following the first SAHC (POST SPIN 1) and second Spin (POST SPIN 2), in addition to before (PRE UPRIGHT), and after upright trunk exercises (POST UPRIGHT).

**Results:**

No significant effect upon IVD height (L2–S1) when performed UPRIGHT or on the SAHC was observed. Trunk muscle exercise induced significant (*p* < 0.05) reduction of active thoracic vertebral compliance when performed on the SAHC, but not UPRIGHT. However, no effect was observed in the cervical, lumbar or across the entire vertebral column. On passive or active vertebral compliance.

**Conclusion:**

This study, the first of its kind demonstrates that trunk exercise were feasible and tolerable. Whilst trunk muscle exercise appears to have minor effect upon IVD height, it may be a candidate approach to mitigate—particularly active—vertebral stability on Earth, and in μg via concurrent SAHC. However, significant variability suggests larger studies including optimization of trunk exercise and SAHC prescription with MRI are warranted.

***Trial Registration*:**

North Rhine ethical committee (Number: 6000223393) and registered on 29/09/2020 in the German Clinical Trials Register (DRKS00021750).

## Introduction

Spinal unloading in microgravity (μg) is associated with stature increments of varying magnitude up to seven cm [1] and transient (up to 4 days) moderate-to-severe (mainly Lumbar) back pain [2] in the majority (53–68%) of astronauts. Whilst the specific pathophysiological mechanisms underlying stature increments and back pain are unknown, spaceflight is associated with intervertebral disc (IVD) changes [3], including swelling [4], trunk muscle atrophy [5], reduced para-spinal muscle tone [6], spinal curvature flattening [7] and impaired spinal kinematics [8]. Such changes may also contribute to increased vertebral column vulnerability that could support an apparent increased risk of IVD herniation [9] event that is debilitating on Earth but could be critical when landing on the Moon.

Some astronauts have, due to increased stature, experienced difficulties fitting into designated extra-vehicular activity (EVA) suits, and prior to returning to Earth, their bespoke Soyuz Kazbek seat pan [9]. Despite the clear operational significance, and significant lumbar IVD pathology being observed with Magnetic Resonance Imaging (MRI) following long duration spaceflight [5], very few spinal evaluation studies have been performed inflight [10]. However, a novel in-flight ultrasonic protocol developed by NASA [11] was employed in seven long-duration astronauts; identifying 14 features of IVD pathology, including disk desiccation and osteophytes not observed pre-flight [12]. However, no significant changes in IVD height or angle were observed [12], despite dynamic Lumbar IVD changes being reported in response to diurnal loading [13], exercise-induced loading [14] and even simple re-orientation [15] on Earth.

Changes in body position [16] or gravitational loading [17] have also been demonstrated to rapidly modulate vertebral stiffness—defined as the vertebral column’s resistance to deformation [18]. Vertebral compliance is posture-dependent [16] with postural muscle activation associated with weight-bearing leading to the term ‘active’ vertebral stiffness when upright, and ‘passive’ when prone and thus non-axial load bearing [19]. In fact, a recent parabolic flight study reported acute increments in lumbar (L3) vertebral compliance during transient (~ 20 s) μg, with comparable reductions in vertebral compliance during hypergravity (~ 1.8 g) when standing ‘upright’[17]. However, the effects of loading associated with exercise in varying gravitational environments is unknown.

Indeed, whilst some of the spinal column changes (or their apparent absence) following long-duration (~ 6 months) missions [5] may reflect gravitational exposure associated with re-entry and landing [10] life on the International Space Station (ISS) does not mean that the spinal column is continuously unloaded [10]. In fact, the in-flight exercise countermeasures [20] intended to ameliorate multi-systems de-conditioning i.e., resistive exercise (Advanced Resistive Exercise Device: ARED) and aerobic training (T2—treadmill) also results in repeated exposure to transient (and potentially high instantaneous) axial loading, even though it does not target the spine, or trunk musculature [10]. Whilst it is likely that such exercise affects IVD geometry and may contribute to IVD pathology and vertebral vulnerability, the effect of exercise in non-1 g gravitational loading is unknown [21].

Despite the extensive time and resources expended by astronauts performing exercise countermeasures, to variable degrees on the ISS [22], significant deconditioning remains an issue, particularly in the musculoskeletal [23], neuro-motor [24], and cardiorespiratory systems [25]. Furthermore, no current in-flight exercise countermeasures targeted maintaining vertebral column function—thus significant post-flight para-spinal muscle atrophy [5, 6] and trunk muscle dysfunction [26] is observed, including exaggerated vertebral stiffness [27]. Thus, development of novel in-flight countermeasures that are not only more effective at mitigating multi-system de-conditioning, in addition to protecting the vertebral column are warranted, despite future spaceflight resources including mass and volume, being more constrained than currently on the ISS [28].

Re-imposition of axial ‘gravitational-like’ forces has been proposed via elasticated body suits [29, 30]. Indeed, ‘SkinSuits’ have been shown to reduce stature on Earth [31], tolerable in μg [32], and whose intermittent donning may promote vertebral column functionality, including IVD geometry by inducing moderate axial reloading [33]. However, such approaches are unlikely to mitigate multi-systems deconditioning. Provision of Artificial Gravity (AG) via short-arm human centrifugation (SAHC) has been proposed as a potential approach to ameliorate multi-systems de-conditioning, including the vertebral column [34].

Data from several short-duration head down bed rest (HDBR) studies (the most common ground-based microgravity analogue) suggest that passive AG exposure may have protective effects on induced musculoskeletal de-conditioning [35] and orthostatic intolerance [36]. Daily passive AG at 1 g at the Centre of Mass (CoM) has also been reported to be both tolerable and acceptable [37]. However, 30 min of daily 1 g at CoM AG provides a low physiological load [38], and thus appears to be ineffective at ameliorating HDBR-induced multi-systems deconditioning [39, 40].

Performance of exercise during AG has been associated with disorientation, motion sickness [41] and orthostatic intolerance [42]. However, when the g load is moderate (i.e., around 1 g at CoM), and body motion is voluntary and head movements are consistent with it—e.g., squatting—movement is well tolerated and motion sickness suppressed [43]. Interestingly, following squatting during AG with 1.5 g at the CoM significant lumbar IVD compression was observed [unpublished observations 44].

On Earth, trunk exercises that activate core stabilizer muscles are prescribed in an attempt to mitigate back pain [45] and promote spinal-related functionality [46]. For example, lateral stabilization and trunk rotation (wood chopper) exercises have been proposed as effective interventions for low back pain [47]. Furthermore, isometric abdominal exercises that activate transversus abdominis (TrA) promote local dynamic spine stability [48]. Yet the effect of such exercise on IVD geometry is unknown. Furthermore, it is reported that the activation of trunk muscles, when supporting loads, can reduce active vertebral compliance [16].

Thus, performance of lateral stabilization, trunk rotation and isometric abdominal exercises during concurrent axial loading induced by AG is a novel candidate approach to address μg—induced back pain and vertebral column dysfunction. However, whether such exercises generate IVD height compression and vertebral compliance modulation consistent with comparable exercise when upright in 1 g is unknown.

## Aim

The aim of the study was to determine the effect of lateral stabilization, trunk rotation and isometric abdominal exercise upon lumbar IVD height, and both passive and active vertebral compliance when performed supine on a SAHC with 1 g at the CoM, compared to that generated with equivalent upright exercise in 1 g.

## Methods

12 (8 male) healthy subjects (33.8 ± 7 years, 178.4 ± 8.2 cm, 72.1 ± 9.6 kg) gave written informed consent to participate in the study approved by the North Rhine ethical committee (Number: 6000223393) and registered on 29/09/2020 in the German Clinical Trials Register (DRKS00021750). Prior to inclusion in the study, all subjects completed a centrifuge medical screening which included blood tests, urine analysis, medical history, and both a resting and treadmill-based stress test ECG. All subjects were recreationally active, including performance of sports-based physical activity at least twice per week.

The study was performed at the short-arm human centrifuge (SAHC) within the: Envihab facility at the German Aerospace Center (DLR) in Cologne (Germany). SAHC is designed for a maximum radial acceleration of 6 g at outer perimeter (i.e., at the feet). During the ramp up/down phases (de)acceleration did not exceed 5°s^−2^ to minimize the risk of vestibular-induced tumbling sensations. On the centrifuge, participants were secured in a supine position on a horizontal sledge system against a fixed footplate and were instructed to avoid unnecessary head movements during centrifugation to minimize the provocation of disorientation/motion sickness symptoms.

**Fig. 1:**
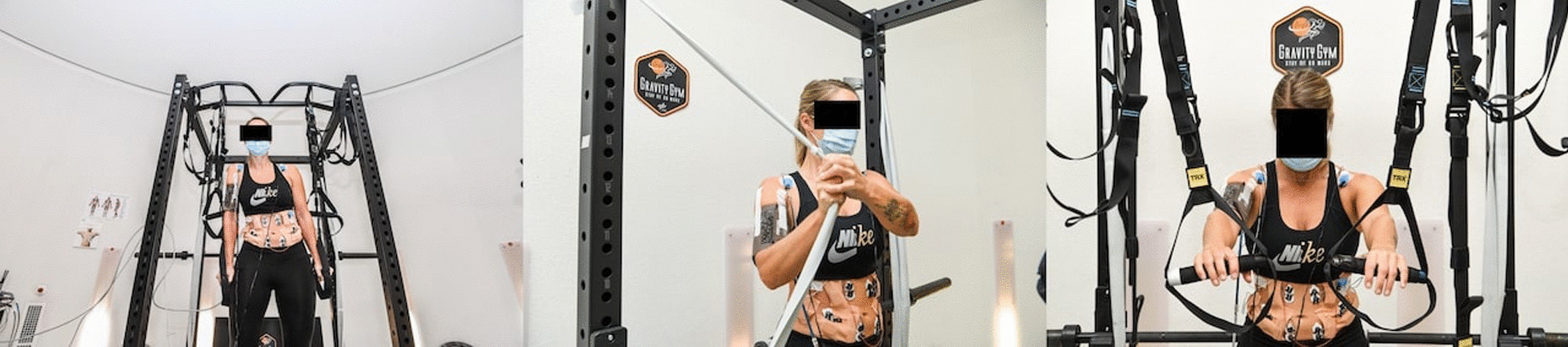
Contralateral (left), Trunk Rotation (Wood Chopper; centre) and Abdominal Isometric exercises (right) performance at 1 g (UPRIGHT)

**Fig. 2:**
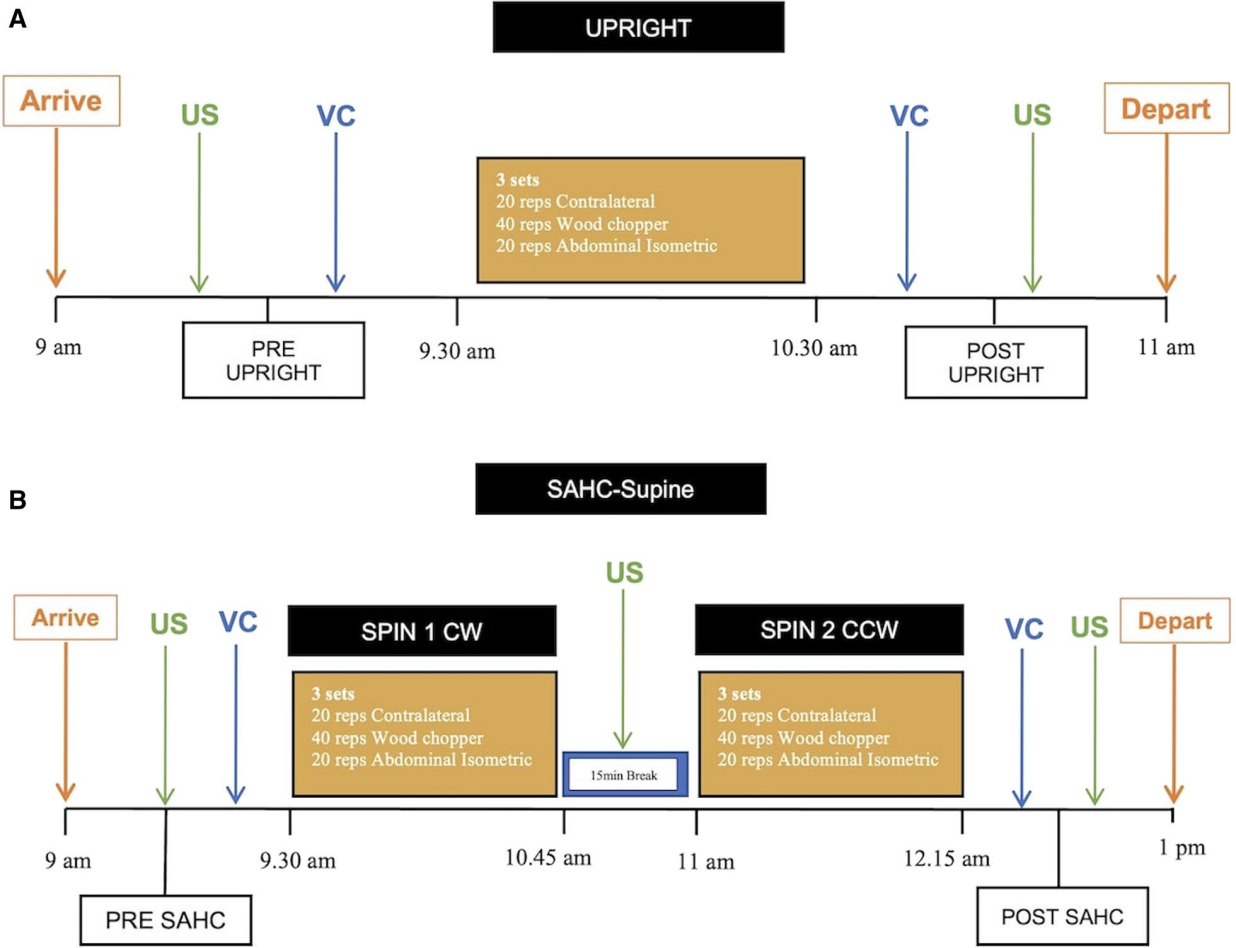
**A** Schematic of the upright (UPRIGHT) protocol with ultrasonic (US) and vertebral compliance (VC) (active (upright) and passive (supine)) measurements obtained before (PRE UPRIGHT), and after upright trunk exercises in 1 g (POST UPRIGHT). **B**. Schematic of the 1 g at the Centre of Mass (CoM) short-arm centrifugation (SAHC) on supine position; protocol with ultrasonic (US) and vertebral compliance (VC) (active (upright) and passive (supine)) measurements obtained before (PRE SAHC), and after SAHC with trunk exercise performance (POST SAHC)

Subjects attended the facility on two occasions, on non-sequential days following the initial medical screening. Each session included performance of three sets of upper body exercise trunk exercises: lateral stabilization (contralateral), trunk rotation (wood chopper) and abdominal isometric when standing upright (UPRIGHT) (Figs. 1, 2a), and during being spun when supine on the SAHC at an angular velocity sufficient to generate 1 g at that individual’s CoM (SAHC) (Fig. 2b) in a randomized order. The SAHC condition consisted of 2 separate centrifuge runs, one clockwise (CW), and the other counter-clockwise (CCW) which were also randomised, although the order of the exercises within each condition was consistent.

In each condition three sets of 20, contralateral exercises (10 each side) were performed, with the arms stretched out holding TRX-bands (TRX Training, USA) while standing one-legged on a balance air pillows (Sissel, Germany), alternating the support leg after each five-second hold. Three sets of 40 (20 each side) wood chopper exercises (involving upper body rotation) with resistance provided by holding resistance bands (TheraBand® 3–4.3 lbs, TheraBand, USA) on each side were also performed. Finally, three sets of 20 abdominal isometric exercises involving a ‘push-down’ movement holding TRX-bands with both hands in front of the abdomen were performed, with each set separated by 60 s of rest.

For the SAHC session, having been familiarised, subjects were instrumented, and lay supine secured (with a hip safety belt) on a sledge, which allowed motion along the SAHC radius with minimal friction. Each subject’s head was orientated towards the centre of rotation with their feet placed on force plates mounted on the end of the centrifuge arm. Subjects lay supine for 5 min (PRE SAHC) on the stationary SAHC, before being spun for 10 min (with 30 s ramp up/down phases) separated by a 15-min break between the two SAHC runs.

Participants were asked to report any back pain, or discomfort in either condition.

Lumbar IVD height and vertebral compliance (when passive (supine) and active (upright)) were evaluated prior to SAHC (PRE SAHC) and following the SPIN 1 (Clockwise; CW). Vertebral compliance was recorded after SPIN 2 (Counter-clockwise; CCW) (POST SAHC) because participants were secured with a hip safety belt and thus were unable to turn over between runs, in addition, to before (PRE UPRIGHT), and after upright trunk exercise performance (POST UPRIGHT).

**Fig. 3:**
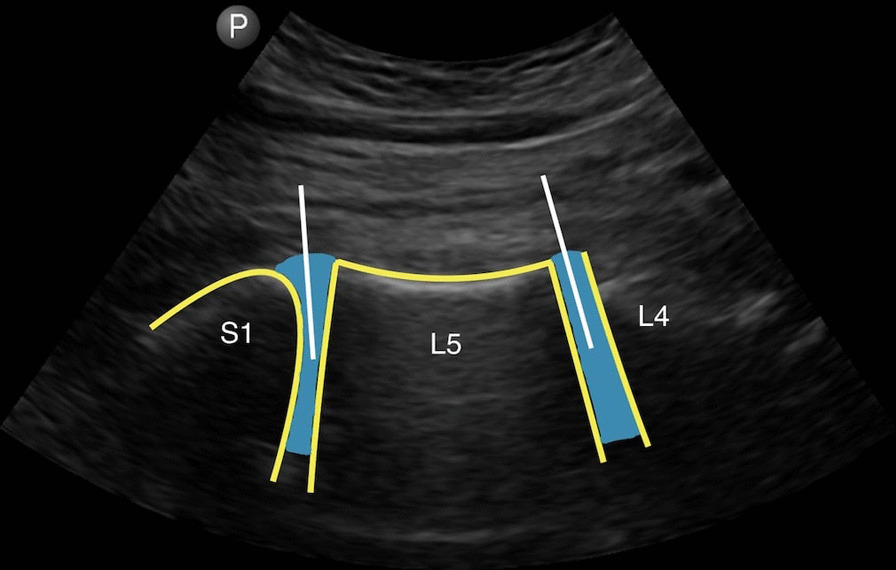
Image of L4–S1 with ultrasonic long-axis scanning

Lumbar IVD height from L1 to S1 (L1–L2, L2–L3, L3–L4, L4–L5, L5–S1) was assessed via portable ultrasound (Lumify, Philips, Netherlands) with a curvilinear array probe (5–15 MHz) connected to a Galaxy S2 tablet (Samsung, South Korea) when lay prone on a clinical couch. At least two images per level were acquired: one high gain and one low gain allowing estimation of respective anterior IVD height (long axis) (Fig. 3).

Vertebral compliance from C1–L5 was assessed in active (when upright) and passive (prone) conditions with a handheld differential vertebral compliance transducer (PulStar, Sense Technology Inc., USA) manually placed and held perpendicularly upon each vertebral spinous process. To trigger the vertebral compliance measurement, a preload of (18 N) [49] was applied to compress the soft tissue components between the transducer head and the target spinous process [49, 50]. The triggered impulse propagation properties reflect vertebral compliance [51] captured via dedicated software (PulStarFRAS, Sense Technology Inc., USA). Resultant vertebral compliance (C1 to L5) is reported to possess good- test-rest reliability even with trained novice examiners [50], with excellent reliability across the spine [52]. Assessment at each spinous process was performed twice and averaged [53].

### Data analysis

All data was normally distributed (Shapiro Wilk’s test). The effect of upright trunk exercise performance upon IVD height (PRE UPRIGHT vs. POST UPRIGHT) in 1 g was evaluated with paired t-tests. The effect trunk exercise upon IVD height during 1 g at CoM generated by SAHC was evaluated by a one-way ANOVA (PRE SAHC, POST SPIN 1 and POST SPIN 2). As no specific effect of SPIN was evident, the mean effect of trunk exercise during SAHC with 1 g at the CoM (ΔPOST SAHC − PRE SAHC) was compared with that generated when performed upright in 1 g (ΔPOST UPRIGHT − PRE UPRIGHT) via paired t-tests.

Passive and active vertebral compliance were compared in the UPRIGHT and SAHC conditions by paired t-tests. The effect of trunk exercise performance upon active and passive vertebral compliance when upright (PRE UPRIGHT vs. POST UPRIGHT) and on the SAHC (PRE SAHC vs POST SPIN 2) was compared across the entire column and each spinal segment (cervical, thoracic and lumbar) by paired t-tests. As there was no additional effect of Spin 2 the overall effect was calculated by deltas (Δ) (POST–PRE). Changes in passive and active vertebral compliance were compared between UPRIGHT and SAHC with paired t-tests.

Data are reported as mean ± standard error of the mean (SEM). All statistical tests were conducted using IBM SPSS version 21 (IBM Corp., USA). *P* < 0.05 was assumed to indicate statistical significance with Hedge’s *g* effect sizes reported to further contextualise the data. Hedge’s g can be used for ‘paired’ effect size calculations when the sample sizes are low, with 0.2, 0.5 and 0.8 being defined as small, medium and large, respectively (based on 1 indicating a group difference equal to 1 standard deviation of the mean) [54].

## Results

All 3 trunk exercises were well tolerated by the subjects in both the UPRIGHT and SAHC 1 g at CoM conditions. No participant reported back pain, or discomfort in either condition. One participant demonstrated pre-syncopal symptoms during SAHC that were mitigated by performance of calf-raises for 1 min under the direction of the supervising physician. No centrifuge run or exercise session was terminated.

### IVD height

IVD image quality was satisfactory except for the level L1–L2, and thus is not reported. No significant difference in IVD height was observed across levels L2 to S1 following trunk muscle exercise performed upright in 1 g (UPRIGHT), although there was a trend (*p* = 0.058) for L2–L3 height reductions with a ‘large’ hedge’s *g* (1.39) effect size (Table 1).

**Table 1:** Mean (± SEM) Intervertebral disc (IVD) (L2–S1) height (cm) before (PRE UPRIGHT) and following upright trunk exercise performance (POST UPRIGHT) in 1 g

IVD level (cm)	PRE UPRIGHT	POST UPRIGHT	Hedge’s *g*	Paired t-test
L2–L3	0.65 ± 0.01	0.58 ± 0.02	1.39	*p* = 0.058
L3–L4	0.75 ± 0.01	0.75 ± 0.03	0.08	*p* = 0.804
L4–L5	0.89 ± 0.02	0.89 ± 0.02	0.03	*p* = 0.928
L5–S1	1.18 ± 0.02	1.14 ± 0.02	0.49	*p* = 0.223

Paired t-tests with Hedge’s *g* effect size calculations were used to compare conditions

No effect of trunk exercise performance during SAHC (with 1 g at the CoM) was observed across L2 to S1 IVD height (Table 2). Thus, the second spin had no additional demonstrable effect.

**Table 2:** Mean (± SEM) Intervertebral disc (IVD) (L2–S1) height (cm) before (PRE SAHC) and following the first (POST SPIN 1) and second (POST SPIN 2) exposure to 1 g at the CoM via short-arm centrifugation (SAHC)

IVD level (cm)	PRE SAHC	POST SPIN 1	POST SPIN 2 (POST SAHC)	One-way ANOVA
L2–L3	0.60 ± 0.01	0.59 ± 0.02	0.59 ± 0.03	[F(2, 14) = 0.268, *p* = 0. 971]
L3–L4	0.76 ± 0.02	0.73 ± 0.01	0.75 ± 0.03	[F(2, 14) = 0.028, *p* = 0.768]
L4–L5	0.83 ± 0.05	0.83 ± 0.06	0.8 ± 0.05	[F(2, 14) = 0.576, *p* = 0.570]
L5–S1	1.28 ± 0.07	1.21 ± 0.06	1.13 ± 0.05	[F(2, 14) = 1.990, *p* = 0.174]

One-way ANOVAs were used to compare conditions

No significant differences in IVD height changes induced by trunk exercise when upright (ΔPOST UPRIGHT − PRE UPRIGHT) vs. SAHC (ΔPOST SAHC − PRE SAHC) were observed (Table 3). However, a greater reduction in L2–L3 IVD height UPRIGHT compared to SAHC had a ‘large’ Hedge’s *g* (0.73) effect size.

**Table 3:** Mean (± SEM) delta (Δ) Intervertebral disc (IVD) (L2–S1) height after performance of trunk muscle exercise when upright in 1 g (ΔPOST—PRE UPRIGHT), and during exposure to 1 g (at the CoM) via short-arm centrifugation (ΔPOST—PRE SAHC)

IVD level (cm)	Δ (POST − PRE) UPRIGHT	Δ(POST − PRE) SAHC	Hedge’s *g*	Paired t-test
L2–L3	− 0.065 ± 0.027	− 0.005 ± 0.027	0.73	*p* = 0.300
L3–L4	− 0.006 ± 0.024	− 0.007 ± 0.045	0.01	*p* = 0.404
L4–L5	0.002 ± 0.026	− 0.037 ± 0.031	0.37	*p* = 0.984
L5–S1	− 0.037 ± 0.028	− 0.157 ± 0.093	0.58	*p* = 0.115

Paired t-tests with Hedge’s *g* effect size calculations were used to compare conditions

### Vertebral compliance

Active vertebral compliance (Newtons) was significantly greater than passive vertebral compliance (with ‘large’ Hedge’s *g* effect sizes) in each spinal segment (cervical, thoracic and lumbar) and across the entire column prior to both conditions (Table 4).

**Table 4:** Mean (± SEM) passive (prone) and active (upright) vertebral compliance (Newtons) across the entire spinal column (C1 to L5) and each segment prior to the performance of upright trunk muscle exercise in 1 g (PRE UPRIGHT) and during exposure to 1 g at the CoM via short-arm centrifugation (PRE SAHC)

Spine level	Passive (PRE UPRIGHT)	Active (PRE UPRIGHT)	Hedge’s *g*	Paired t-test UPRIGHT	Passive (PRE SAHC)	Active (PRE SAHC)	Hedge’s *g*	Paired t-test SAHC
Entire column	77.6 ± 1.7	91.0 ± 1.7	2.61	*p < 0.01	81.0 ± 1.2	92.0 ± 1.6	2.59	*p < 0.01
Cervical	77.3 ± 2.2	87.5 ± 1.9	1.64	*p < 0.01	79.3 ± 1.01	87.2 ± 1.4	2.11	*p = 0.01
Thoracic	77.4 ± 1.5	93.5 ± 1.7	3.33	*p < 0.01	81.0 ± 1.8	95.4 ± 1.7	2.7	*p < 0.01
Lumbar	78.1 ± 2.5	90.0 ± 1.7	1.80	*p < 0.01	83.4 ± 2.5	91.3 ± 2.8	0.96	*p < 0.01
Upper lumbar	79.5 ± 2.3	91.7 ± 2.0	1.86	*p < 0.01	84.9 ± 2.6	95.2 ± 2.4	1,33	*p = 0.04
Lower lumbar	79.6 ± 2.3	87.3 ± 1.7	1.24	*p < 0.01	81.21 ± 2.4	87.8 ± 2.2	0.95	*p* = 0.13

Paired t-tests with Hedge’s *g* effect size calculations were used to compare conditions. *Indicates significant (*p* < 0.05) difference between passive and active postures

Trunk muscle exercise during UPRIGHT and SAHC failed to induce significant reductions in passive vertebral compliance in the entire column, cervical, thoracic, or lumbar regions (Table 5). However, UPRIGHT trunk exercise induced reduced passive vertebral compliance with ‘large’ Hedge’s *g* effect sizes in all regions (and entire column), except for the lumbar region.

**Table 5:** Mean (± SEM) passive (prone) vertebral compliance (Newtons) across the entire spinal column (C1 to L5) and each segment prior to (PRE UPRIGHT) and following (POST UPRIGHT) the performance of trunk muscle exercise in 1 g; and prior to (PRE SAHC) and following (POST SAHC) the performance trunk muscle exercise during 1 g at the CoM via short-arm centrifugation

Spine level	Passive PRE UPRIGHT	Passive POST UPRIGHT	Hedge’s *g*	Paired t-test UPRIGHT	Passive PRE SAHC	Passive POST SAHC (SPIN 2)	Hedge’s *g*	Paired t-test SAHC
Entire column	77.5 ± 1.7	74.0 ± 0.7	0.89	*p* = 0.064	81.0 ± 1.2	80.6 ± 1.1	0.11	*p* = 0.756
Cervical	77.3 ± 2.2	72.4 ± 1.4	0.87	*p* = 0.091	79.3 ± 1.0	79.4 ± 0.7	0.02	*p* = 0.936
Thoracic	77.4 ± 1.5	73.4 ± 1.1	0.98	*p* = 0.084	81.0 ± 1.8	80.2 ± 1.9	0.14	*p* = 0.708
Lumbar	78.1 ± 2.5	75.1 ± 2.2	0.41	*p* = 0.299	83.4 ± 2.5	83.3 ± 1.6	0.02	*p* = 0.945
Upper lumbar	77.1 ± 1.7	74.8 ± 1.9	0.32	*p* = 0.494	84.9 ± 2.6	83.3 ± 1.7	0.24	*p* = 0.542
Lower lumbar	79.5 ± 2.3	75.6 ± 3.1	0.47	*p* = 0.145	81.2 ± 2.4	83.1 ± 2.0	0.29	*p* = 0.508

Paired t-tests with Hedge’s *g* effect size calculations were used to compare conditions

UPRIGHT trunk muscle exercise induced significant reductions (with ‘medium’ Hedge’s *g* effect sizes) in thoracic, but not cervical, lumbar, or entire column active vertebral compliance (Table 6). Similarly, significant reductions in thoracic active vertebral compliance were induced (with ‘large’ Hedge’s *g* effect sizes) by trunk muscle exercise during SAHC, but not in the cervical, lumbar, or entire column.

**Table 6:** Mean (± SEM) passive (prone) vertebral compliance (Newtons) across the entire spinal column (C1 to L5) and each segment prior to (PRE SAHC) and following (POST SAHC) the performance of trunk muscle exercise during 1 g at the CoM via short-arm centrifugation

Spine level	Active PRE UPRIGHT	Active POST UPRIGHT	Hedge’s *g*	Paired T-Test UPRIGHT	Active PRE SAHC	Active POST SAHC (SPIN 2)	Hedge’s *g*	Paired T-Test SAHC
Entire column	91.0 ± 1.6	89.5 ± 1.7	0.27	*p* = 0.091	92.0 ± 1.6	89.6 ± 1.5	0.50	*p* = 0.150
Cervical	87.5 ± 1.9	84.8 ± 1.6	0.49	*p* = 0.068	87.2 ± 1.4	84.9 ± 1.9	0.45	*p* = 0.145
Thoracic	93.5 ± 1.7	91.1 ± 1.9	0.44	*p = 0.035	95.4 ± 1.7	91.4 ± 1.7	0.79	*p = 0.043
Lumbar	90.0 ± 1.8	90.3 ± 2.5	0.04	*p* = 0.758	91.3 ± 2.9	89.1 ± 2.0	0.30	*p* = 0.511
Upper lumbar	91.7 ± 2.0	93.0 ± 2.6	0.18	*p* = 0.297	95.2 ± 2.4	93.3 ± 1.9	0.28	*p* = 0.537
Lower lumbar	87.34 ± 1.7	88.1 ± 2.1	0.13	*p* = 0.549	87.8 ± 2.2	86.4 ± 2.3	0.21	*p* = 0.549

Paired t-tests with Hedge’s *g* effect size calculations were used to compare conditions. *Indicates significant (*p* < 0.05) difference between PRE and POST conditions

No significant differences (or strong Hedge’s *g*) were observed Δ (POST–PRE) between UPRIGHT and SAHC in passive or active vertebral compliance over the entire column, cervical, thoracic or lumbar segments.

## Discussion

The present study sought to determine the effect of lateral stabilization, trunk rotation and isometric abdominal exercises upon lumbar IVD height, and both passive and active vertebral compliance when performed supine on a SAHC with 1 g at the CoM, compared to that generated with equivalent upright exercise in 1 g.

The main findings of the study were that trunk (lateral stabilization, trunk rotation and isometric abdominal) exercises were feasible and tolerable but had no significant effect upon IVD height (L2–S1) when performed upright or supine on the SAHC with 1 g at the CoM. However, a ‘large’ hedge’s *g* effect size was observed for L2–L3 compression following UPRIGHT trunk exercise. Active vertebral compliance was significantly greater than passive across the entire column and in each spinal (cervical, thoracic and lumbar) segment prior to both UPRIGHT and SAHC trunk muscle exercise. Trunk muscle exercise failed to induce significant reduction of passive vertebral compliance when performed UPRIGHT, although ‘large’ Hedge’s *g* effect sizes were observed in all but the lumbar region*.* UPRIGHT and SAHC trunk muscle exercise induced significant reductions in thoracic, but not cervical, lumbar, or entire column active vertebral compliance with no differences between conditions.

No reports of discomfort, pain or motion sickness were observed in either condition, suggesting that the performance of lateral stabilization, trunk rotation and isometric abdominal exercises is compatible with SAHC with 1 g at the CoM.

### Effects on intervertebral disc height

Trunk (lateral stabilization, trunk rotation and isometric abdominal) exercises had no effect upon IVD height (L2–S1) when performed upright, or supine on the SAHC with 1 g at the CoM. However, following UPRIGHT trunk exercise reduction of L2–L3 height had a ‘large’ hedge’s *g* effect size. In addition, a ‘large’ hedge’s *g* effect size was reported for L2–L3 IVD height reductions POST SAHC compared to UPRIGHT. This suggests that the lower lumbar region appears most sensitive and that loading during SAHC was less effective than UPRIGHT.

Interestingly, IVD compression—in particular lower lumbar—has been observed following re-orientation [15], diurnal loading on Earth [13] and exercise-induced loading [14, 55]. However, this data was acquired with MRI, which is more sensitive and less subject to investigator error than ultrasound. Our data was captured with ultrasound as MRI is currently not compatible with spaceflight. Whilst this study suggests functionally significant IVD compression was not induced, we have previously observed significant disc compression following squatting during SAHC with 1 g, and to a greater extent 1.5 g at the CoM [unpublished observations 44]. Thus, follow up studies with MRI are warranted.

The magnitude of IVD compression may depend on the muscle volume pressure exerted on the vertebral column [56]. For example, an axial load ≥ 45% of body weight can lead to spinal motor control changes capable of modulating lumbar geometry [18]. However, the muscle volume pressure exerted on the vertebral column associated with our trunk muscle exercise is presumably low and broadly distributed [57]. Indeed, IVD compression has been shown to depend upon loading characteristics including direction, frequency magnitude and duration [21] with axial loading and exercise shown to provide compression forces that significantly differ between segments and vertebra [52].

Interestingly, we failed to observed compression in the larger lower IVDs (e.g., L5–S1) which are considered more sensitive to acute (gravitational) loading [unpublished observation 44, 58]. This is expressed in the L5–S1 disc possessing the lowest proteoglycan content, and thus lowest swelling pressure [59]. Indeed, posture has been demonstrated to possess a dramatic effect on IVD pressures [60] with Wilke et al. reporting IVD pressures of 0.1 Mpa when lying prone, and 0.5 when standing [61]. Such measures may inform modelling of potential effects upon IVD hydration and/or protein content.

Intervertebral aging is associated by dehydration and protein depletion [62–64] and thus an increased risk of IVD herniation similar to chronic spinal unloading [65, 66]. In fact, whilst long term HDBR has been shown to increase spinal length and IVD area, with reduced IVD angles, 30 min daily passive SAHC with 1 g at the CoM had no significant effect [67], similar to other musculoskeletal parameters [39, 40]. Whether trunk muscle exercise is more effective is unknown, however core-strengthening exercise has been shown to remodel IVD content, with repeated loading, such as that experienced by athletes associated with increased glycosaminoglycan content [68] in contrast to reduced content observed in individuals with IVD pathology on Earth [69] and in space [3].

Observed IVD compression was minor, but this coupled with changes in spinal column curvature [7] may have induced changes in stature. Unfortunately, stature could not be measured accurately on the SAHC. However, whilst SAHC at 1 g at the CoM is potentially compatible with exploration missions [28] it appears insufficient to mitigate extra-vehicular activity (EVA) suit donning, and Soyuz Kazbek seat pan fit issues [9] associated with μg-induced stature increments [1].

### Effects on vertebral compliance

Active vertebral compliance was significantly greater than passive vertebral compliance across the entire column and in each spinal (cervical, thoracic and lumbar) segment prior to both UPRIGHT and SAHC trunk muscle exercise. This finding is consistent with previous reports when moving from prone to upright [70] as biomechanical changes including increased trunk muscle tensions and IVD pressures lead to elevated spinal stiffness [71].

In our study, trunk muscle exercise failed to induce significant reduction of passive vertebral compliance when performed UPRIGHT. However, ‘large’ Hedge’s *g* were observed across the entire column, the cervical and thoracic regions, but not lumbar. No such effects were observed during SAHC with 1 g at the CoM suggesting that whilst a greater number of subjects may have yielded significant reductions when UPRIGHT, no such effect is likely with 1 g SAHC. Differences in spinal control are evident in vitro [19] compared with in vivo [18]. In vitro studies test passive structural elements such as connective tissue compliance tension, but neglect muscle activity—a critical contributor to net vertebral stabilisation [17]. Therefore, passive vertebral compliance tending not to reduce as markedly as active vertebral compliance suggests that evaluation of the dynamics of trunk muscle contributions must be considered [16].

UPRIGHT and SAHC trunk muscle exercise induced significant acute reduction in thoracic, but not cervical, lumbar, or entire column active vertebral compliance, with no differences between conditions. In fact, reductions in active vertebral compliance are consistent with those reported when donning a backpack [16] or via axial loading [18], and during a recent parabolic flight where vertebral compliance reductions were observed during hypergravity (~ 1.8 g) when standing ‘upright’ [17]. Our set of exercises were determined to target activation of vertebral stabilizers [72], therefore their activation is likely to redistribute load [73], potentially decreasing vertebral stiffness [74]. However, this hypothesis needs further research as a significant inter-subject variability in vertebral compliance measures has been observed [50], a potential confound given the relatively low sample size of the current study.

Cyclic flexion–extension in lumbar viscoelastic tissues also induces vertebral compliance reduction [75]. However, the failure to observe significant effects by lateral stabilization, trunk rotation and isometric abdominal exercises may be due to the fact that they predominantly recruit muscles operating upon the thoracic vertebrae. Indeed, further evaluation of the specific effect of candidate exercises is warranted to inform definition of exercise prescriptions targeted at ameliorating acute vertebral control issues [27] and impaired spinal kinematics [8]—at least of the thoracic vertebrae—with, and without SAHC.

## Conclusion

This study, the first of its kind demonstrates that trunk (lateral stabilization, trunk rotation and isometric abdominal) exercises were feasible and tolerable but had no significant effect upon IVD height (L2–S1) when performed upright or supine on the SAHC with 1 g at the CoM identifiable by ultrasound. However, ‘large’ hedge’s *g* effect sizes were observed for UPRIGHT induced L2–L3 reductions. Active vertebral compliance was significantly greater than passive across the entire column and in each spinal (cervical, thoracic and lumbar) segment prior to both UPRIGHT and SAHC trunk muscle exercise. Trunk muscle exercise failed to induce significant reduction of passive vertebral compliance when performed on the SAHC and UPRIGHT, although ‘large’ Hedge’s *g* effect sizes were observed in all but the lumbar region for UPRIGHT*.* UPRIGHT and SAHC trunk muscle exercise induced significant reductions in thoracic, but not cervical, lumbar, or entire column active vertebral compliance with no differences between conditions. Thus, whilst trunk muscle exercise appears to have minor effect upon IVD height, it may be a candidate approach to mitigate—particularly active—vertebral stability on Earth, and in μg via concurrent SAHC. However, significant variability suggests larger studies including optimization of trunk exercise and SAHC prescription with MRI are warranted—ideally following protracted unloading.

## Data Availability

The datasets generated during and/or analysed during the current study are available from the corresponding author on reasonable request.

## Abbreviations

ARED: Advanced Resistive Exercise Device
TrA: Activate transversus abdominis
AG: Artificial Gravity
CW: Clockwise
CCW: Counter-clockwise
DLR: German Aerospace Center
IVD: Intervertebral disc
CoM: Centre of mass
SAHC: Short-arm human centrifugation
HDBR: Short-duration head down bed rest
